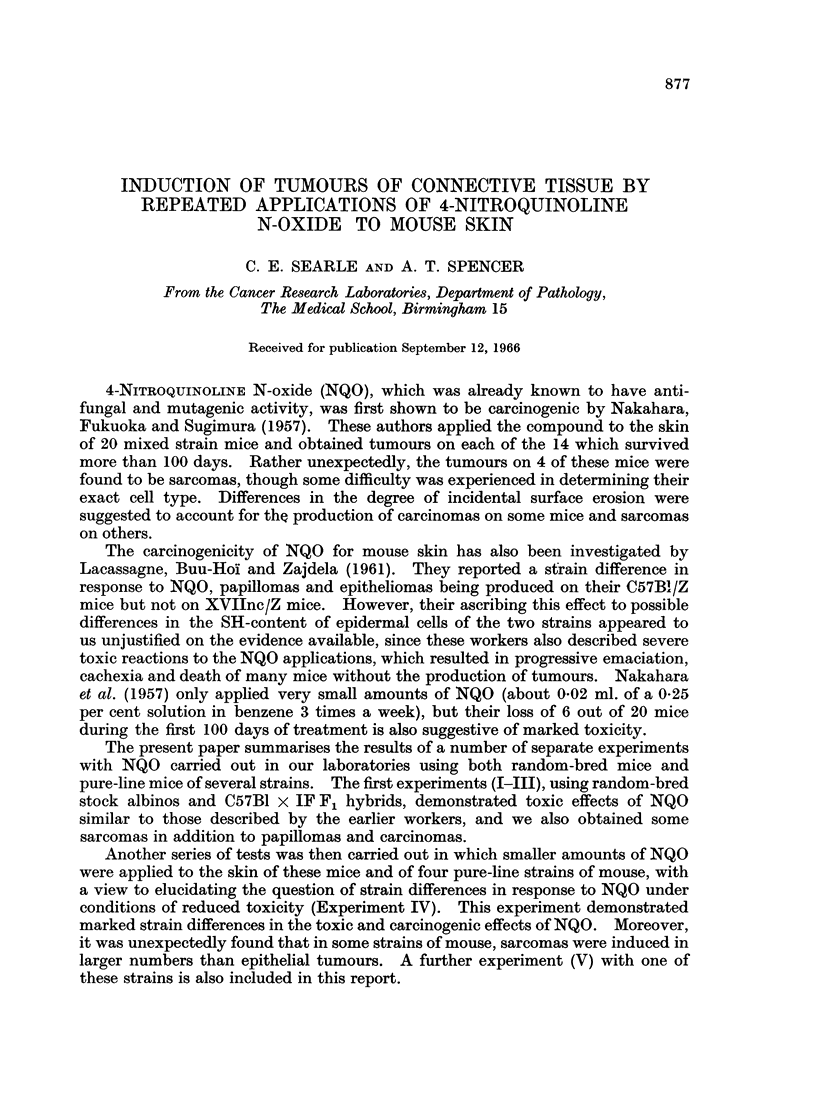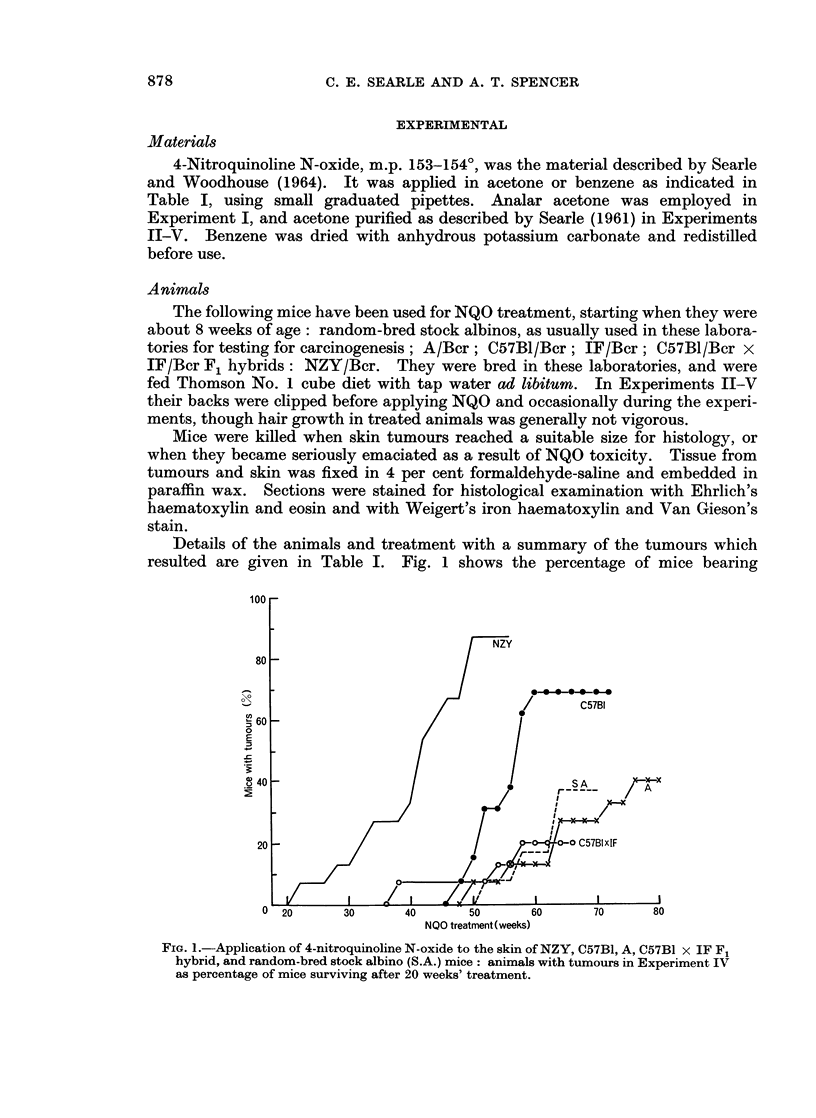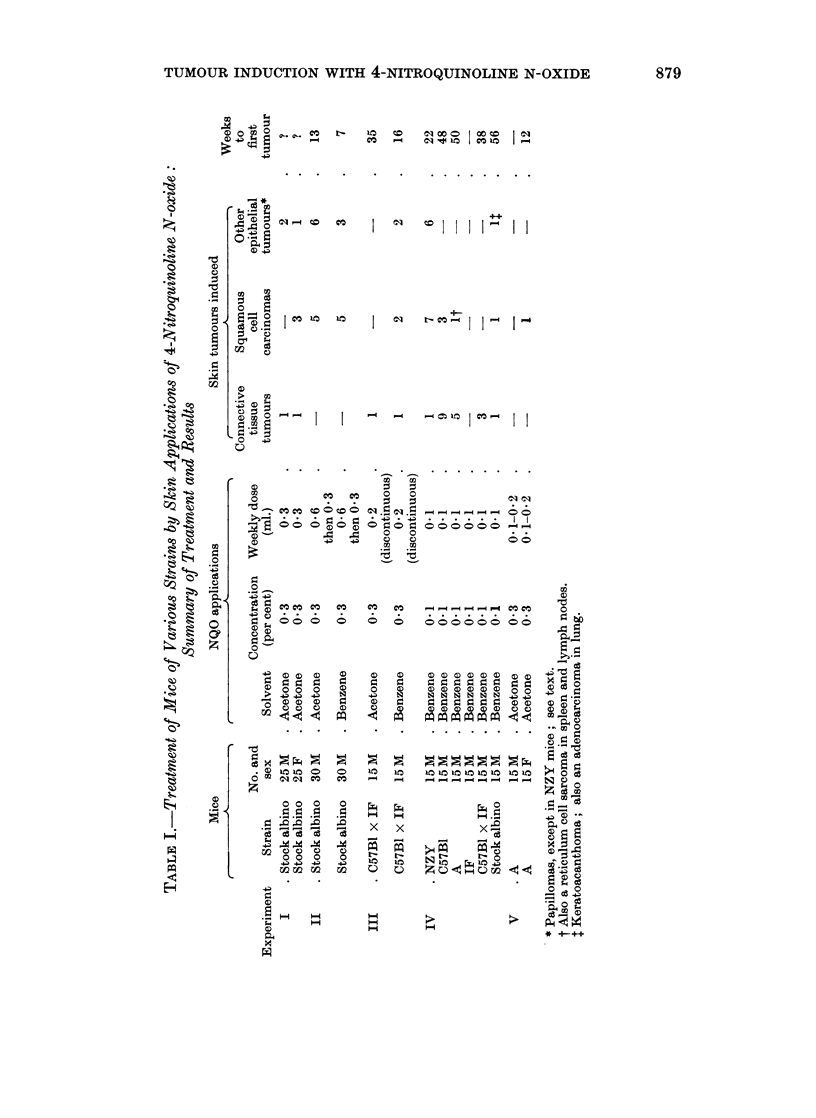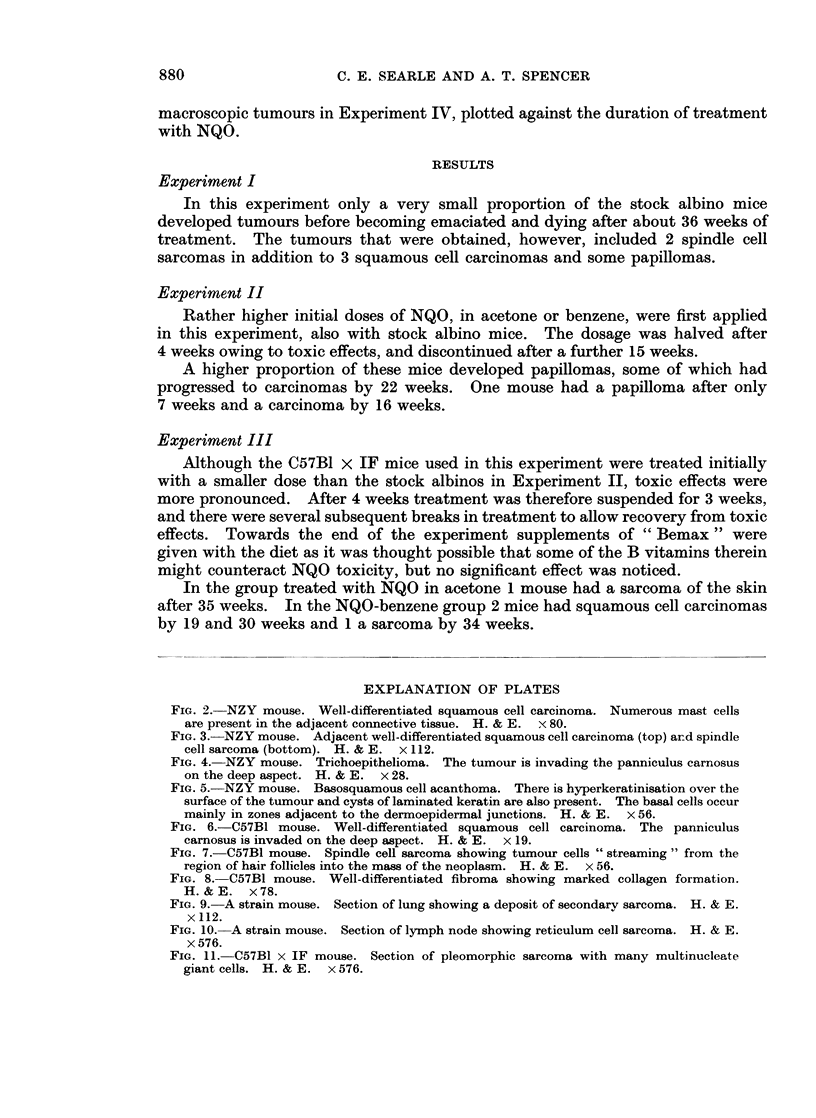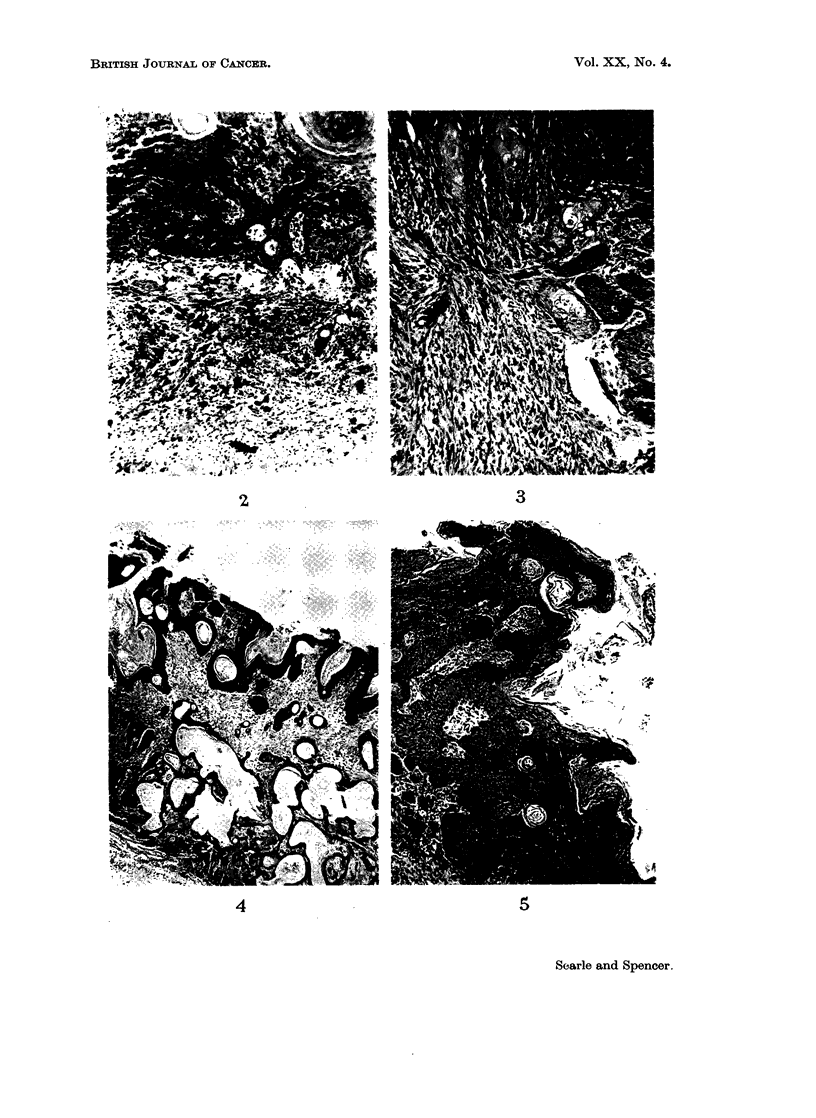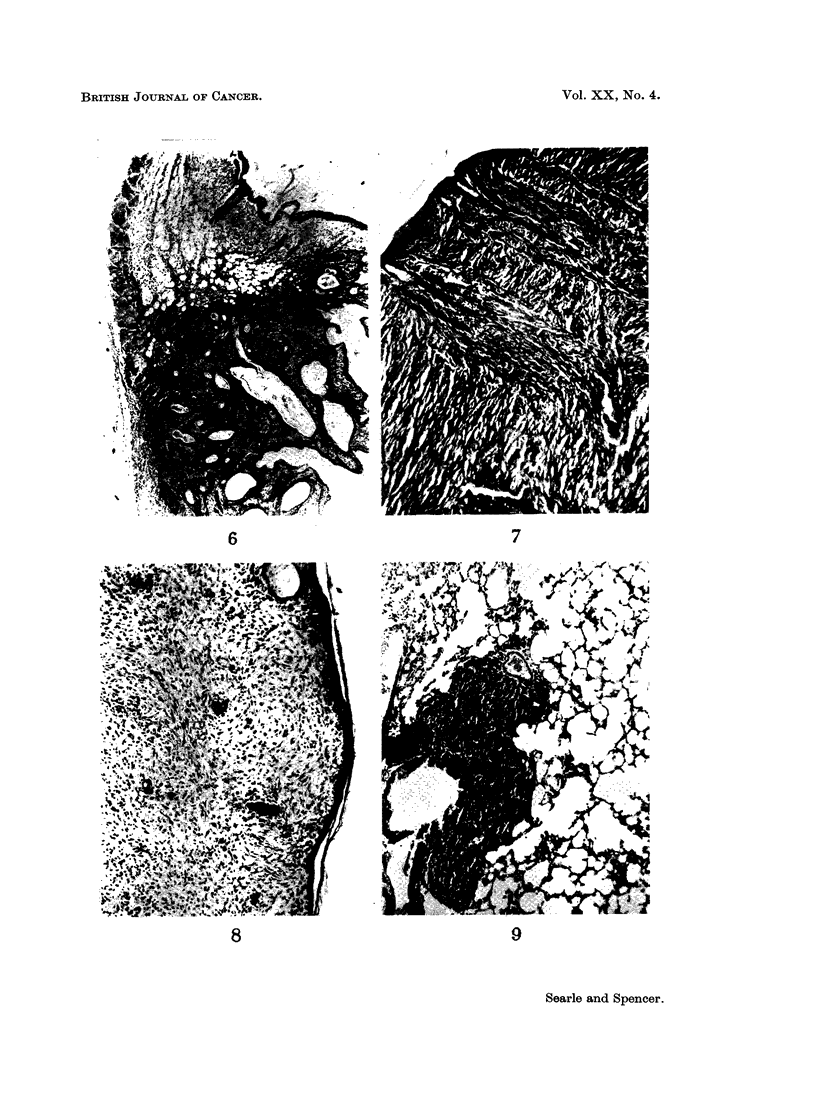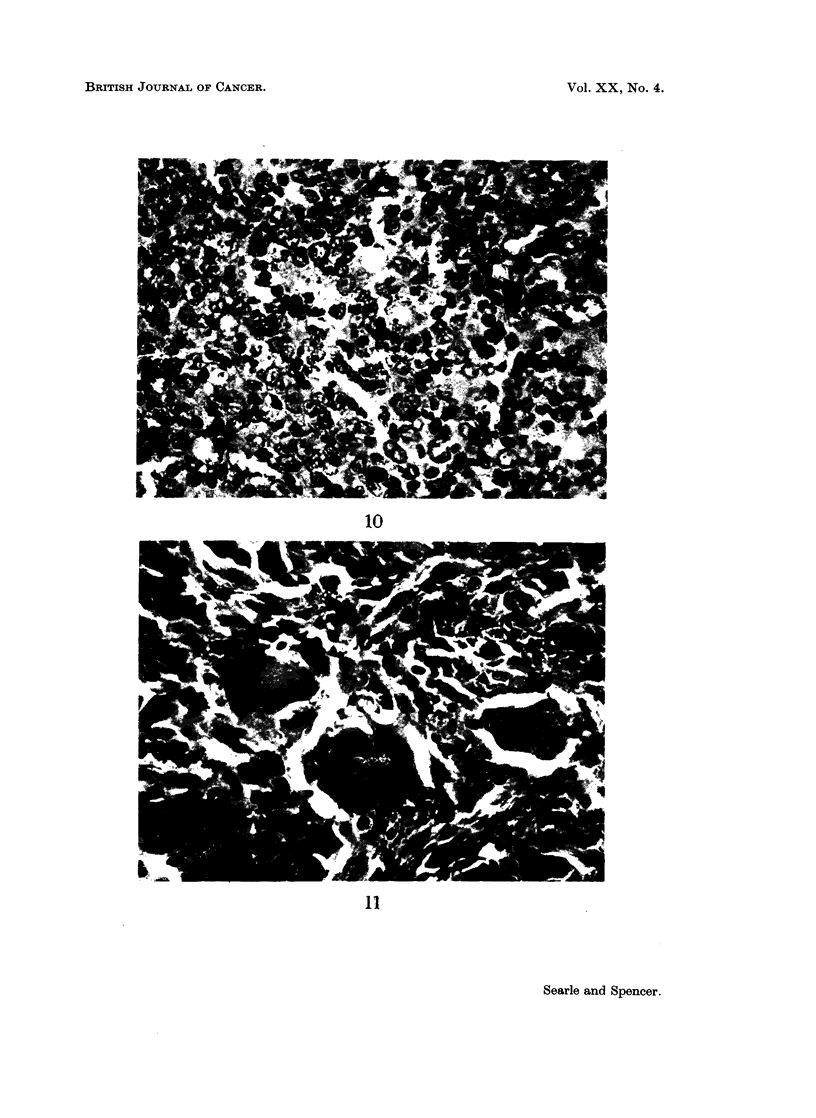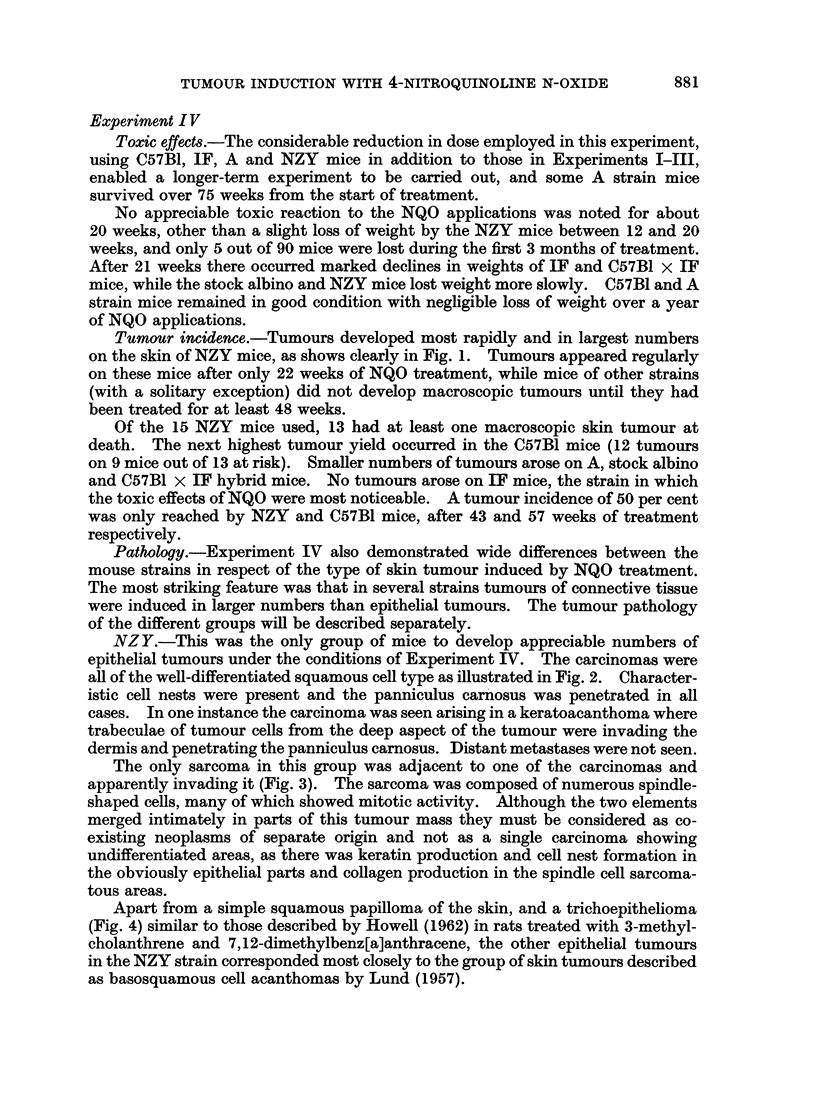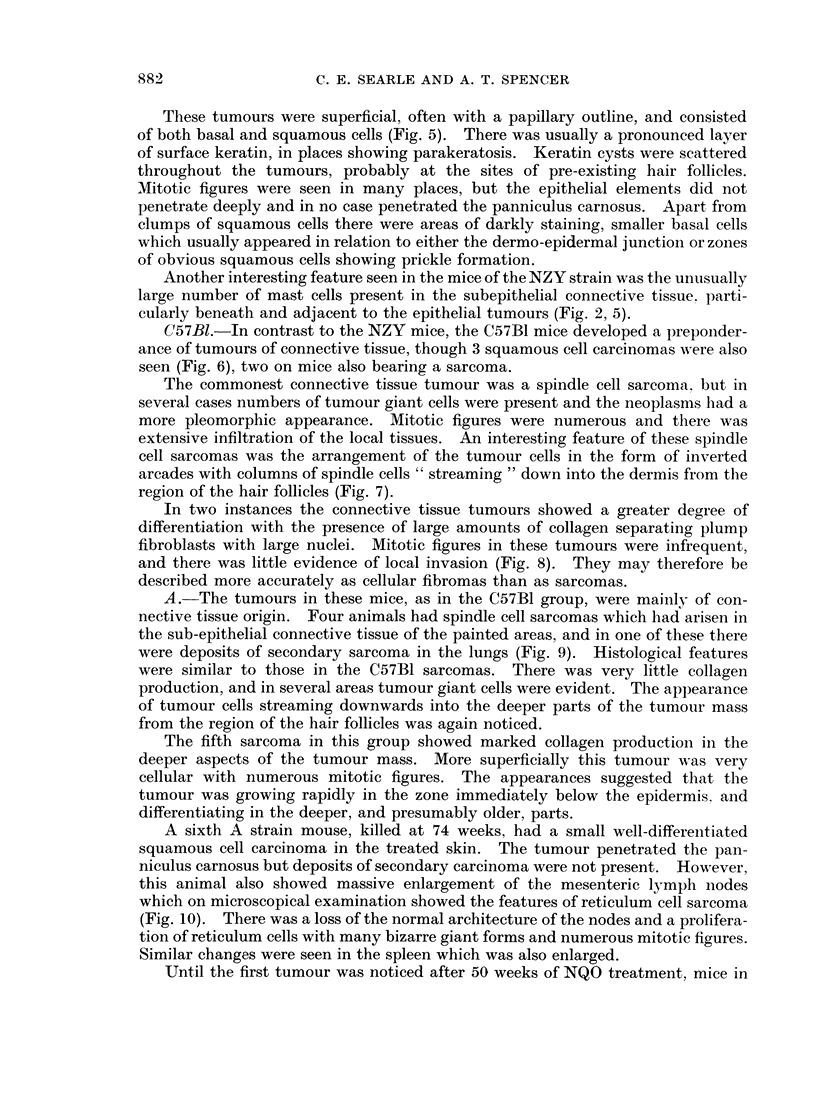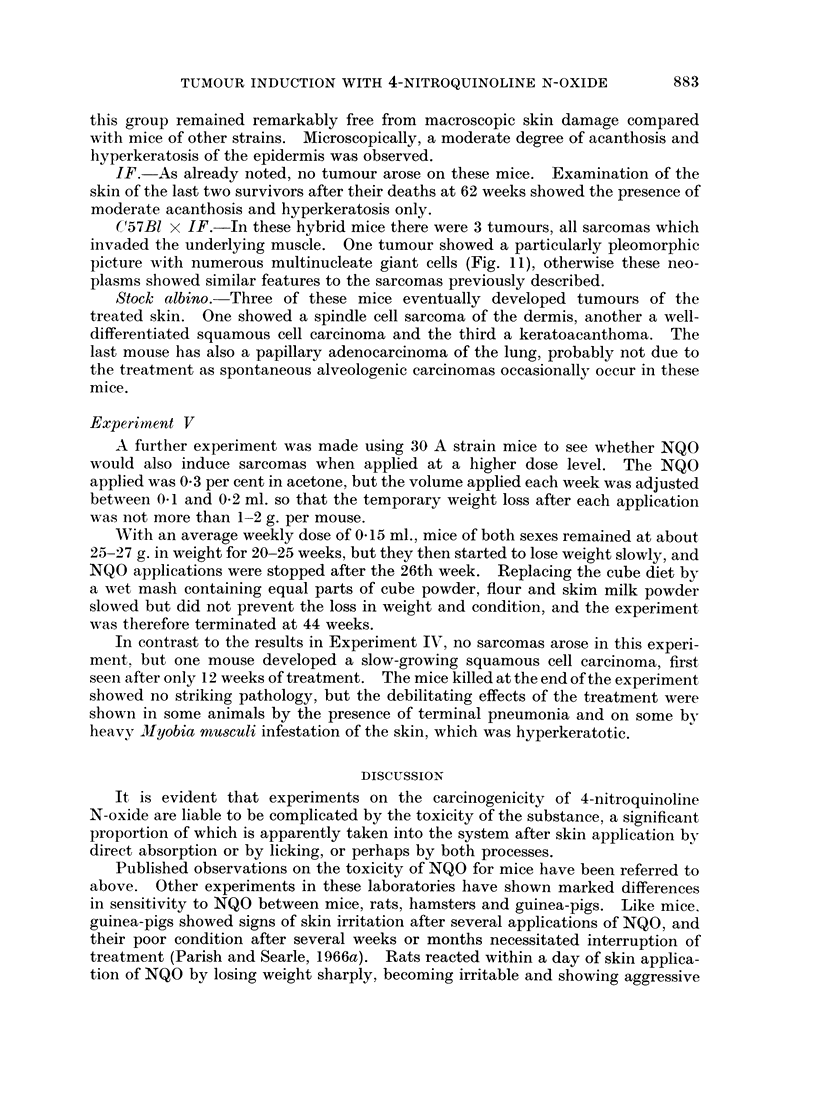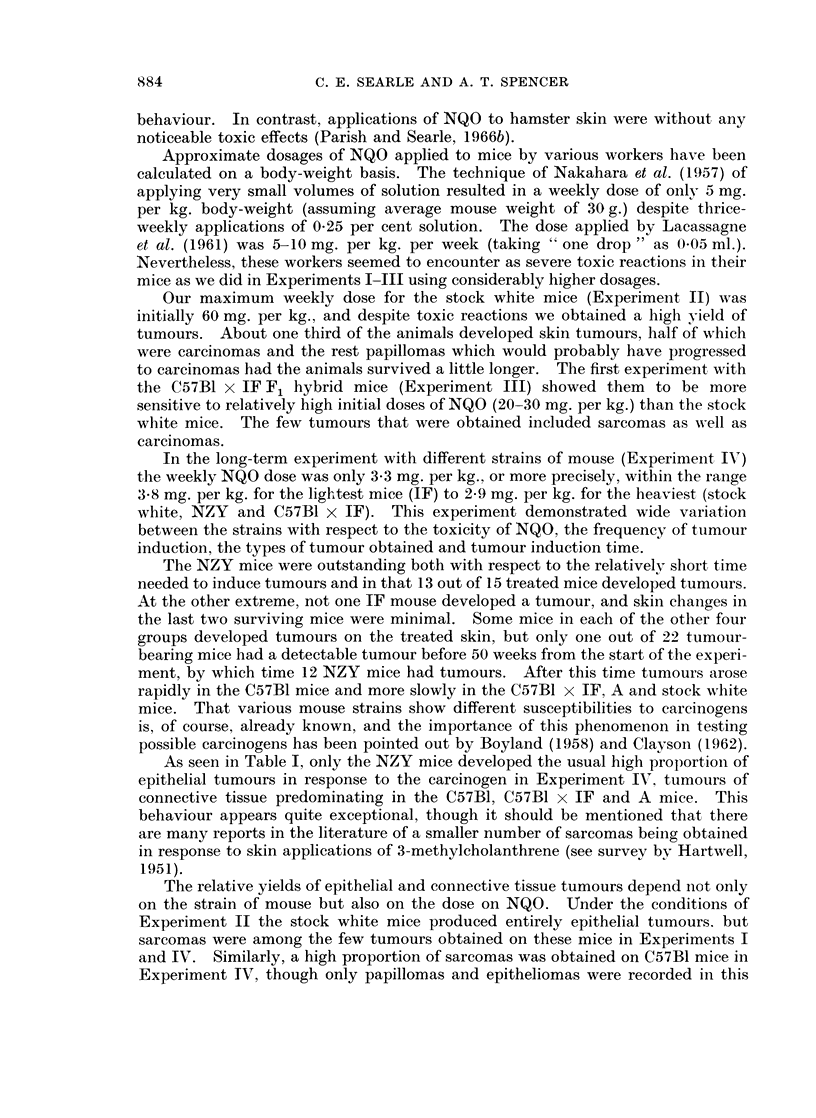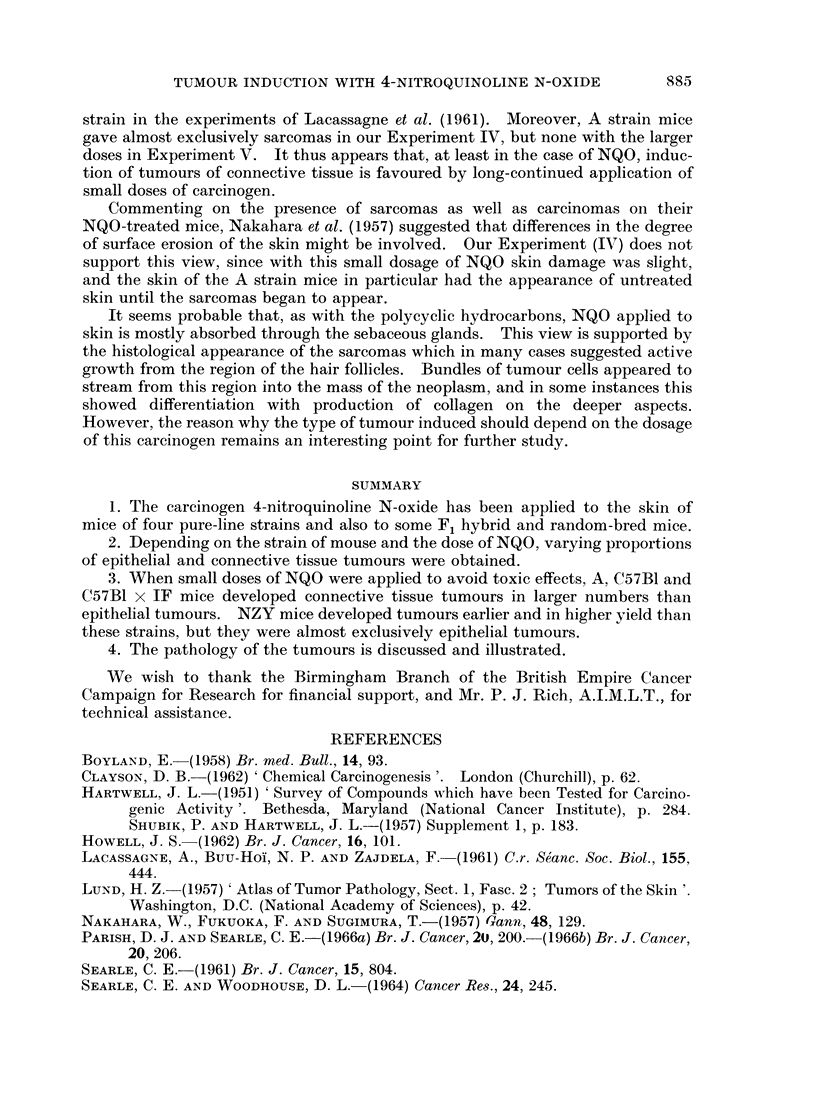# Induction of tumours of connective tissue by repeated applications of 4-nitroquinoline N-oxide to mouse skin.

**DOI:** 10.1038/bjc.1966.101

**Published:** 1966-12

**Authors:** C. E. Searle, A. T. Spencer

## Abstract

**Images:**


### INDUCTION OF TUMOURS OF CONNECTIVE TISSUE BY REPEATED APPLICATIONS OF 4-NITROQUINOLINE N-OXIDE TO MOUSE SKIN

C. E. SEARLE AND A. T. SPENCER

*From the Cancer Research Laboratories, Department of Pathology, The Medical School, Birmingham* 15

Received for publication September 12, 1966

4-NITROQUINOLINE N-oxide (NQO), which was already known to have antifungal and mutagenic activity, was first shown to be carcinogenic by Nakahara, Fukuoka and Sugimura (1957). These authors applied the compound to the skin of 20 mixed strain mice and obtained tumours on each of the 14 which survived more than 100 days. Rather unexpectedly, the tumours on 4 of these mice were found to be sarcomas, though some difficulty was experienced in determining their exact cell type. Differences in the degree of incidental surface erosion were suggested to account for the production of carcinomas on some mice and sarcomas on others.

The carcinogenicity of NQO for mouse skin has also been investigated by Lacassagne, Buu-Hoï and Zajdela (1961). They reported a strain difference in response to NQO, papillomas and epitheliomas being produced on their C57Bl/Z mice but not on XVIInc/Z mice. However, their ascribing this effect to possible differences in the SH-content of epidermal cells of the two strains appeared to us unjustified on the evidence available, since these workers also described severe toxic reactions to the NQO applications, which resulted in progressive emaciation, cachexia and death of many mice without the production of tumours. Nakahara *et al.* (1957) only applied very small amounts of NQO (about 0·02 ml. of a 0·25 per cent solution in benzene 3 times a week), but their loss of 6 out of 20 mice during the first 100 days of treatment is also suggestive of marked toxicity.

The present paper summarises the results of a number of separate experiments with NQO carried out in our laboratories using both random-bred mice and pure-line mice of several strains. The first experiments (I–III), using random-bred stock albinos and C57Bl × IF $F_1$ hybrids, demonstrated toxic effects of NQO similar to those described by the earlier workers, and we also obtained some sarcomas in addition to papillomas and carcinomas.

Another series of tests was then carried out in which smaller amounts of NQO were applied to the skin of these mice and of four pure-line strains of mouse, with a view to elucidating the question of strain differences in response to NQO under conditions of reduced toxicity (Experiment IV). This experiment demonstrated marked strain differences in the toxic and carcinogenic effects of NQO. Moreover, it was unexpectedly found that in some strains of mouse, sarcomas were induced in larger numbers than epithelial tumours. A further experiment (V) with one of these strains is also included in this report.

### EXPERIMENTAL

### *Materials*

4-Nitroquinoline N-oxide, m.p. 153–154°, was the material described by Searle and Woodhouse (1964). It was applied in acetone or benzene as indicated in Table I, using small graduated pipettes. Analar acetone was employed in Experiment I, and acetone purified as described by Searle (1961) in Experiments II–V. Benzene was dried with anhydrous potassium carbonate and redistilled before use.

### *Animals*

The following mice have been used for NQO treatment, starting when they were about 8 weeks of age : random-bred stock albinos, as usually used in these laboratories for testing for carcinogenesis ; A/Bcr ; C57Bl/Bcr ; IF/Bcr ; C57Bl/Bcr × IF/Bcr $F_1$ hybrids : NZY/Bcr. They were bred in these laboratories, and were fed Thomson No. 1 cube diet with tap water *ad libitum.* In Experiments II–V their backs were clipped before applying NQO and occasionally during the experiments, though hair growth in treated animals was generally not vigorous.

Mice were killed when skin tumours reached a suitable size for histology, or when they became seriously emaciated as a result of NQO toxicity. Tissue from tumours and skin was fixed in 4 per cent formaldehyde-saline and embedded in paraffin wax. Sections were stained for histological examination with Ehrlich's haematoxylin and eosin and with Weigert's iron haematoxylin and Van Gieson's stain.

Details of the animals and treatment with a summary of the tumours which resulted are given in Table I. Fig. 1 shows the percentage of mice bearing

FIG. 1.—Application of 4-nitroquinoline N-oxide to the skin of NZY, C57Bl, A, C57Bl × IF $F_1$ hybrid, and random-bred stock albino (S.A.) mice : animals with tumours in Experiment IV as percentage of mice surviving after 20 weeks' treatment.

TABLE I.—*Treatment of Mice of Various Strains by Skin Applications of 4-Nitroquinoline N-oxide : Summary of Treatment and Results*

| | Mice | | NQO applications | | | Skin tumours induced | | | |
|---|---|---|---|---|---|---|---|---|---|
| Experiment | Strain | No. and sex | Solvent | Concentration (per cent) | Weekly dose (ml.) | Connective tissue tumours | Squamous cell carcinomas | Other epithelial tumours* | Weeks to first tumour |
| I | Stock albino | 25 M | Acetone | 0·3 | 0·3 | 1 | — | 2 | ? |
| | Stock albino | 25 F | Acetone | 0·3 | 0·3 | 1 | 3 | 1 | ? |
| II | Stock albino | 30 M | Acetone | 0·3 | 0·6 then 0·3 | — | 5 | 6 | 13 |
| | Stock albino | 30 M | Benzene | 0·3 | 0·6 then 0·3 | — | 5 | 3 | 7 |
| III | C57Bl × IF | 15 M | Acetone | 0·3 | 0·2 (discontinuous) | 1 | — | — | 35 |
| | C57Bl × IF | 15 M | Benzene | 0·3 | 0·2 (discontinuous) | 1 | 2 | 2 | 16 |
| IV | NZY | 15 M | Benzene | 0·1 | 0·1 | 1 | 7 | 6 | 22 |
| | C57Bl | 15 M | Benzene | 0·1 | 0·1 | 9 | 3 | — | 48 |
| | A | 15 M | Benzene | 0·1 | 0·1 | 5 | 1† | — | 50 |
| | IF | 15 M | Benzene | 0·1 | 0·1 | — | — | — | — |
| | C57Bl × IF | 15 M | Benzene | 0·1 | 0·1 | 3 | — | — | 38 |
| | Stock albino | 15 M | Benzene | 0·1 | 0·1 | 1 | 1 | 1‡ | 56 |
| V | A | 15 M | Acetone | 0·3 | 0·1–0·2 | — | — | — | — |
| | A | 15 F | Acetone | 0·3 | 0·1–0·2 | — | 1 | — | 12 |

* Papillomas, except in NZY mice ; see text.

† Also a reticulum cell sarcoma in spleen and lymph nodes.

‡ Keratoacanthoma ; also an adenocarcinoma in lung.

macroscopic tumours in Experiment IV, plotted against the duration of treatment with NQO.

### RESULTS

### *Experiment I*

In this experiment only a very small proportion of the stock albino mice developed tumours before becoming emaciated and dying after about 36 weeks of treatment. The tumours that were obtained, however, included 2 spindle cell sarcomas in addition to 3 squamous cell carcinomas and some papillomas.

### *Experiment II*

Rather higher initial doses of NQO, in acetone or benzene, were first applied in this experiment, also with stock albino mice. The dosage was halved after 4 weeks owing to toxic effects, and discontinued after a further 15 weeks.

A higher proportion of these mice developed papillomas, some of which had progressed to carcinomas by 22 weeks. One mouse had a papilloma after only 7 weeks and a carcinoma by 16 weeks.

### *Experiment III*

Although the C57Bl × IF mice used in this experiment were treated initially with a smaller dose than the stock albinos in Experiment II, toxic effects were more pronounced. After 4 weeks treatment was therefore suspended for 3 weeks, and there were several subsequent breaks in treatment to allow recovery from toxic effects. Towards the end of the experiment supplements of " Bemax " were given with the diet as it was thought possible that some of the B vitamins therein might counteract NQO toxicity, but no significant effect was noticed.

In the group treated with NQO in acetone 1 mouse had a sarcoma of the skin after 35 weeks. In the NQO-benzene group 2 mice had squamous cell carcinomas by 19 and 30 weeks and 1 a sarcoma by 34 weeks.

---

### EXPLANATION OF PLATES

FIG. 2.—NZY mouse. Well-differentiated squamous cell carcinoma. Numerous mast cells are present in the adjacent connective tissue. H. & E. ×80.

FIG. 3.—NZY mouse. Adjacent well-differentiated squamous cell carcinoma (top) and spindle cell sarcoma (bottom). H. & E. ×112.

FIG. 4.—NZY mouse. Trichoepithelioma. The tumour is invading the panniculus carnosus on the deep aspect. H. & E. ×28.

FIG. 5.—NZY mouse. Basosquamous cell acanthoma. There is hyperkeratinisation over the surface of the tumour and cysts of laminated keratin are also present. The basal cells occur mainly in zones adjacent to the dermoepidermal junctions. H. & E. ×56.

FIG. 6.—C57Bl mouse. Well-differentiated squamous cell carcinoma. The panniculus carnosus is invaded on the deep aspect. H. & E. ×19.

FIG. 7.—C57Bl mouse. Spindle cell sarcoma showing tumour cells " streaming " from the region of hair follicles into the mass of the neoplasm. H. & E. ×56.

FIG. 8.—C57Bl mouse. Well-differentiated fibroma showing marked collagen formation. H. & E. ×78.

FIG. 9.—A strain mouse. Section of lung showing a deposit of secondary sarcoma. H. & E. ×112.

FIG. 10.—A strain mouse. Section of lymph node showing reticulum cell sarcoma. H. & E. ×576.

FIG. 11.—C57Bl × IF mouse. Section of pleomorphic sarcoma with many multinucleate giant cells. H. & E. ×576.

2

3

4

5

Searle and Spencer.

6

7

8

9

Searle and Spencer.

10

11

*Experiment IV*

*Toxic effects.*—The considerable reduction in dose employed in this experiment, using C57Bl, IF, A and NZY mice in addition to those in Experiments I–III, enabled a longer-term experiment to be carried out, and some A strain mice survived over 75 weeks from the start of treatment.

No appreciable toxic reaction to the NQO applications was noted for about 20 weeks, other than a slight loss of weight by the NZY mice between 12 and 20 weeks, and only 5 out of 90 mice were lost during the first 3 months of treatment. After 21 weeks there occurred marked declines in weights of IF and C57Bl × IF mice, while the stock albino and NZY mice lost weight more slowly. C57Bl and A strain mice remained in good condition with negligible loss of weight over a year of NQO applications.

*Tumour incidence.*—Tumours developed most rapidly and in largest numbers on the skin of NZY mice, as shows clearly in Fig. 1. Tumours appeared regularly on these mice after only 22 weeks of NQO treatment, while mice of other strains (with a solitary exception) did not develop macroscopic tumours until they had been treated for at least 48 weeks.

Of the 15 NZY mice used, 13 had at least one macroscopic skin tumour at death. The next highest tumour yield occurred in the C57Bl mice (12 tumours on 9 mice out of 13 at risk). Smaller numbers of tumours arose on A, stock albino and C57Bl × IF hybrid mice. No tumours arose on IF mice, the strain in which the toxic effects of NQO were most noticeable. A tumour incidence of 50 per cent was only reached by NZY and C57Bl mice, after 43 and 57 weeks of treatment respectively.

*Pathology.*—Experiment IV also demonstrated wide differences between the mouse strains in respect of the type of skin tumour induced by NQO treatment. The most striking feature was that in several strains tumours of connective tissue were induced in larger numbers than epithelial tumours. The tumour pathology of the different groups will be described separately.

*NZY.*—This was the only group of mice to develop appreciable numbers of epithelial tumours under the conditions of Experiment IV. The carcinomas were all of the well-differentiated squamous cell type as illustrated in Fig. 2. Characteristic cell nests were present and the panniculus carnosus was penetrated in all cases. In one instance the carcinoma was seen arising in a keratoacanthoma where trabeculae of tumour cells from the deep aspect of the tumour were invading the dermis and penetrating the panniculus carnosus. Distant metastases were not seen.

The only sarcoma in this group was adjacent to one of the carcinomas and apparently invading it (Fig. 3). The sarcoma was composed of numerous spindle-shaped cells, many of which showed mitotic activity. Although the two elements merged intimately in parts of this tumour mass they must be considered as co-existing neoplasms of separate origin and not as a single carcinoma showing undifferentiated areas, as there was keratin production and cell nest formation in the obviously epithelial parts and collagen production in the spindle cell sarcomatous areas.

Apart from a simple squamous papilloma of the skin, and a trichoepithelioma (Fig. 4) similar to those described by Howell (1962) in rats treated with 3-methylcholanthrene and 7,12-dimethylbenz[a]anthracene, the other epithelial tumours in the NZY strain corresponded most closely to the group of skin tumours described as basosquamous cell acanthomas by Lund (1957).

These tumours were superficial, often with a papillary outline, and consisted of both basal and squamous cells (Fig. 5). There was usually a pronounced layer of surface keratin, in places showing parakeratosis. Keratin cysts were scattered throughout the tumours, probably at the sites of pre-existing hair follicles. Mitotic figures were seen in many places, but the epithelial elements did not penetrate deeply and in no case penetrated the panniculus carnosus. Apart from clumps of squamous cells there were areas of darkly staining, smaller basal cells which usually appeared in relation to either the dermo-epidermal junction or zones of obvious squamous cells showing prickle formation.

Another interesting feature seen in the mice of the NZY strain was the unusually large number of mast cells present in the subepithelial connective tissue, particularly beneath and adjacent to the epithelial tumours (Fig. 2, 5).

*C57Bl.*—In contrast to the NZY mice, the C57Bl mice developed a preponderance of tumours of connective tissue, though 3 squamous cell carcinomas were also seen (Fig. 6), two on mice also bearing a sarcoma.

The commonest connective tissue tumour was a spindle cell sarcoma, but in several cases numbers of tumour giant cells were present and the neoplasms had a more pleomorphic appearance. Mitotic figures were numerous and there was extensive infiltration of the local tissues. An interesting feature of these spindle cell sarcomas was the arrangement of the tumour cells in the form of inverted arcades with columns of spindle cells " streaming " down into the dermis from the region of the hair follicles (Fig. 7).

In two instances the connective tissue tumours showed a greater degree of differentiation with the presence of large amounts of collagen separating plump fibroblasts with large nuclei. Mitotic figures in these tumours were infrequent, and there was little evidence of local invasion (Fig. 8). They may therefore be described more accurately as cellular fibromas than as sarcomas.

*A.*—The tumours in these mice, as in the C57Bl group, were mainly of connective tissue origin. Four animals had spindle cell sarcomas which had arisen in the sub-epithelial connective tissue of the painted areas, and in one of these there were deposits of secondary sarcoma in the lungs (Fig. 9). Histological features were similar to those in the C57Bl sarcomas. There was very little collagen production, and in several areas tumour giant cells were evident. The appearance of tumour cells streaming downwards into the deeper parts of the tumour mass from the region of the hair follicles was again noticed.

The fifth sarcoma in this group showed marked collagen production in the deeper aspects of the tumour mass. More superficially this tumour was very cellular with numerous mitotic figures. The appearances suggested that the tumour was growing rapidly in the zone immediately below the epidermis, and differentiating in the deeper, and presumably older, parts.

A sixth A strain mouse, killed at 74 weeks, had a small well-differentiated squamous cell carcinoma in the treated skin. The tumour penetrated the panniculus carnosus but deposits of secondary carcinoma were not present. However, this animal also showed massive enlargement of the mesenteric lymph nodes which on microscopical examination showed the features of reticulum cell sarcoma (Fig. 10). There was a loss of the normal architecture of the nodes and a proliferation of reticulum cells with many bizarre giant forms and numerous mitotic figures. Similar changes were seen in the spleen which was also enlarged.

Until the first tumour was noticed after 50 weeks of NQO treatment, mice in this group remained remarkably free from macroscopic skin damage compared with mice of other strains. Microscopically, a moderate degree of acanthosis and hyperkeratosis of the epidermis was observed.

*IF*.—As already noted, no tumour arose on these mice. Examination of the skin of the last two survivors after their deaths at 62 weeks showed the presence of moderate acanthosis and hyperkeratosis only.

*C57Bl* × *IF*.—In these hybrid mice there were 3 tumours, all sarcomas which invaded the underlying muscle. One tumour showed a particularly pleomorphic picture with numerous multinucleate giant cells (Fig. 11), otherwise these neoplasms showed similar features to the sarcomas previously described.

*Stock albino*.—Three of these mice eventually developed tumours of the treated skin. One showed a spindle cell sarcoma of the dermis, another a well-differentiated squamous cell carcinoma and the third a keratoacanthoma. The last mouse has also a papillary adenocarcinoma of the lung, probably not due to the treatment as spontaneous alveologenic carcinomas occasionally occur in these mice.

### *Experiment V*

A further experiment was made using 30 A strain mice to see whether NQO would also induce sarcomas when applied at a higher dose level. The NQO applied was 0·3 per cent in acetone, but the volume applied each week was adjusted between 0·1 and 0·2 ml. so that the temporary weight loss after each application was not more than 1–2 g. per mouse.

With an average weekly dose of 0·15 ml., mice of both sexes remained at about 25–27 g. in weight for 20–25 weeks, but they then started to lose weight slowly, and NQO applications were stopped after the 26th week. Replacing the cube diet by a wet mash containing equal parts of cube powder, flour and skim milk powder slowed but did not prevent the loss in weight and condition, and the experiment was therefore terminated at 44 weeks.

In contrast to the results in Experiment IV, no sarcomas arose in this experiment, but one mouse developed a slow-growing squamous cell carcinoma, first seen after only 12 weeks of treatment. The mice killed at the end of the experiment showed no striking pathology, but the debilitating effects of the treatment were shown in some animals by the presence of terminal pneumonia and on some by heavy *Myobia musculi* infestation of the skin, which was hyperkeratotic.

### DISCUSSION

It is evident that experiments on the carcinogenicity of 4-nitroquinoline N-oxide are liable to be complicated by the toxicity of the substance, a significant proportion of which is apparently taken into the system after skin application by direct absorption or by licking, or perhaps by both processes.

Published observations on the toxicity of NQO for mice have been referred to above. Other experiments in these laboratories have shown marked differences in sensitivity to NQO between mice, rats, hamsters and guinea-pigs. Like mice, guinea-pigs showed signs of skin irritation after several applications of NQO, and their poor condition after several weeks or months necessitated interruption of treatment (Parish and Searle, 1966*a*). Rats reacted within a day of skin application of NQO by losing weight sharply, becoming irritable and showing aggressive behaviour. In contrast, applications of NQO to hamster skin were without any noticeable toxic effects (Parish and Searle, 1966*b*).

Approximate dosages of NQO applied to mice by various workers have been calculated on a body-weight basis. The technique of Nakahara *et al.* (1957) of applying very small volumes of solution resulted in a weekly dose of only 5 mg. per kg. body-weight (assuming average mouse weight of 30 g.) despite thrice-weekly applications of 0·25 per cent solution. The dose applied by Lacassagne *et al.* (1961) was 5–10 mg. per kg. per week (taking "one drop" as 0·05 ml.). Nevertheless, these workers seemed to encounter as severe toxic reactions in their mice as we did in Experiments I–III using considerably higher dosages.

Our maximum weekly dose for the stock white mice (Experiment II) was initially 60 mg. per kg., and despite toxic reactions we obtained a high yield of tumours. About one third of the animals developed skin tumours, half of which were carcinomas and the rest papillomas which would probably have progressed to carcinomas had the animals survived a little longer. The first experiment with the C57Bl × IF $F_1$ hybrid mice (Experiment III) showed them to be more sensitive to relatively high initial doses of NQO (20–30 mg. per kg.) than the stock white mice. The few tumours that were obtained included sarcomas as well as carcinomas.

In the long-term experiment with different strains of mouse (Experiment IV) the weekly NQO dose was only 3·3 mg. per kg., or more precisely, within the range 3·8 mg. per kg. for the lightest mice (IF) to 2·9 mg. per kg. for the heaviest (stock white, NZY and C57Bl × IF). This experiment demonstrated wide variation between the strains with respect to the toxicity of NQO, the frequency of tumour induction, the types of tumour obtained and tumour induction time.

The NZY mice were outstanding both with respect to the relatively short time needed to induce tumours and in that 13 out of 15 treated mice developed tumours. At the other extreme, not one IF mouse developed a tumour, and skin changes in the last two surviving mice were minimal. Some mice in each of the other four groups developed tumours on the treated skin, but only one out of 22 tumour-bearing mice had a detectable tumour before 50 weeks from the start of the experiment, by which time 12 NZY mice had tumours. After this time tumours arose rapidly in the C57Bl mice and more slowly in the C57Bl × IF, A and stock white mice. That various mouse strains show different susceptibilities to carcinogens is, of course, already known, and the importance of this phenomenon in testing possible carcinogens has been pointed out by Boyland (1958) and Clayson (1962).

As seen in Table I, only the NZY mice developed the usual high proportion of epithelial tumours in response to the carcinogen in Experiment IV, tumours of connective tissue predominating in the C57Bl, C57Bl × IF and A mice. This behaviour appears quite exceptional, though it should be mentioned that there are many reports in the literature of a smaller number of sarcomas being obtained in response to skin applications of 3-methylcholanthrene (see survey by Hartwell, 1951).

The relative yields of epithelial and connective tissue tumours depend not only on the strain of mouse but also on the dose on NQO. Under the conditions of Experiment II the stock white mice produced entirely epithelial tumours, but sarcomas were among the few tumours obtained on these mice in Experiments I and IV. Similarly, a high proportion of sarcomas was obtained on C57Bl mice in Experiment IV, though only papillomas and epitheliomas were recorded in this strain in the experiments of Lacassagne *et al.* (1961). Moreover, A strain mice gave almost exclusively sarcomas in our Experiment IV, but none with the larger doses in Experiment V. It thus appears that, at least in the case of NQO, induction of tumours of connective tissue is favoured by long-continued application of small doses of carcinogen.

Commenting on the presence of sarcomas as well as carcinomas on their NQO-treated mice, Nakahara *et al.* (1957) suggested that differences in the degree of surface erosion of the skin might be involved. Our Experiment (IV) does not support this view, since with this small dosage of NQO skin damage was slight, and the skin of the A strain mice in particular had the appearance of untreated skin until the sarcomas began to appear.

It seems probable that, as with the polycyclic hydrocarbons, NQO applied to skin is mostly absorbed through the sebaceous glands. This view is supported by the histological appearance of the sarcomas which in many cases suggested active growth from the region of the hair follicles. Bundles of tumour cells appeared to stream from this region into the mass of the neoplasm, and in some instances this showed differentiation with production of collagen on the deeper aspects. However, the reason why the type of tumour induced should depend on the dosage of this carcinogen remains an interesting point for further study.

### SUMMARY

1. The carcinogen 4-nitroquinoline N-oxide has been applied to the skin of mice of four pure-line strains and also to some $F_1$ hybrid and random-bred mice.

2. Depending on the strain of mouse and the dose of NQO, varying proportions of epithelial and connective tissue tumours were obtained.

3. When small doses of NQO were applied to avoid toxic effects, A, C57Bl and C57Bl $\times$ IF mice developed connective tissue tumours in larger numbers than epithelial tumours. NZY mice developed tumours earlier and in higher yield than these strains, but they were almost exclusively epithelial tumours.

4. The pathology of the tumours is discussed and illustrated.

We wish to thank the Birmingham Branch of the British Empire Cancer Campaign for Research for financial support, and Mr. P. J. Rich, A.I.M.L.T., for technical assistance.

### REFERENCES

BOYLAND, E.—(1958) *Br. med. Bull.*, **14**, 93.

CLAYSON, D. B.—(1962) ‘ Chemical Carcinogenesis ’. London (Churchill), p. 62.

HARTWELL, J. L.—(1951) ‘ Survey of Compounds which have been Tested for Carcinogenic Activity ’. Bethesda, Maryland (National Cancer Institute), p. 284. SHUBIK, P. AND HARTWELL, J. L.—(1957) Supplement 1, p. 183.

HOWELL, J. S.—(1962) *Br. J. Cancer*, **16**, 101.

LACASSAGNE, A., BUU-HOÏ, N. P. AND ZAJDELA, F.—(1961) *C.r. Séanc. Soc. Biol.*, **155**, 444.

LUND, H. Z.—(1957) ‘ Atlas of Tumor Pathology, Sect. 1, Fasc. 2 ; Tumors of the Skin ’. Washington, D.C. (National Academy of Sciences), p. 42.

NAKAHARA, W., FUKUOKA, F. AND SUGIMURA, T.—(1957) *Gann*, **48**, 129.

PARISH, D. J. AND SEARLE, C. E.—(1966*a*) *Br. J. Cancer*, **20**, 200.—(1966*b*) *Br. J. Cancer*, **20**, 206.

SEARLE, C. E.—(1961) *Br. J. Cancer*, **15**, 804.

SEARLE, C. E. AND WOODHOUSE, D. L.—(1964) *Cancer Res.*, **24**, 245.